# Optimizing the diagnosis and treatment of depression in primary care: the emerging role of vortioxetine treatment

**DOI:** 10.3389/fpsyt.2025.1568777

**Published:** 2025-07-01

**Authors:** José Ángel Alcalá, Verónica Olmo Dorado, Guadalupe del Pilar Arilla Herrera, Silvia López Chamón, Vicente Gasull Molinera

**Affiliations:** ^1^ Psychiatry Department, Hospital Universitario Reina Sofía, Córdoba, Spain; ^2^ Family and Community Medicine, Centro de Salud Torreblanca, Seville, Spain; ^3^ Family and Community Medicine, Centro de Salud Alagón, Zaragoza, Spain; ^4^ Family and Community Medicine, Centro de Salud Huerta de los Frailes, Madrid, Spain; ^5^ Family and Community Medicine, Clínica Eliana, Torrent, Valencia, Spain

**Keywords:** major depressive disorder, primary care, vortioxetine, antidepressants, cognitive deficits, emotional blunting

## Abstract

The management of patients with major depressive disorder (MDD) in primary care (PC) represents a significant challenge and a great opportunity for early and effective intervention. Primary care physicians, as first-line physicians, play a key role in the identification, diagnosis and initial treatment of depression, often being the first and sometimes the only point of contact for these patients with the healthcare system. In this context, the search for effective and well-tolerated therapeutic strategies is constant, and vortioxetine represents a good pharmacological option within the therapeutic armamentarium available to the PC physician. This article explores best practices in the management of MDD from the PC perspective, addressing the specific challenges faced by these professionals and examining the potential role of vortioxetine in the treatment of different patient profiles.

## Introduction

Major depressive disorder (MDD) is characterized by a heterogeneous set of symptoms involving emotional, physical and cognitive impairment (1). The overall burden of MDD has increased in recent years, particularly since 2020, associated with the aftermath of the COVID-19 pandemic (2). According to data from the World Health Organization (WHO), it is estimated that 5% of all adults worldwide suffer depression (6% of women and 4% of men) and 5.7% of all adults over 60 years of age (3). With these data, primary care (PC) is fundamental in the detection, diagnosis, treatment and follow-up of major depressive disorder.

## Complexity of the diagnosis of MDD in primary care

**Figure 1:**
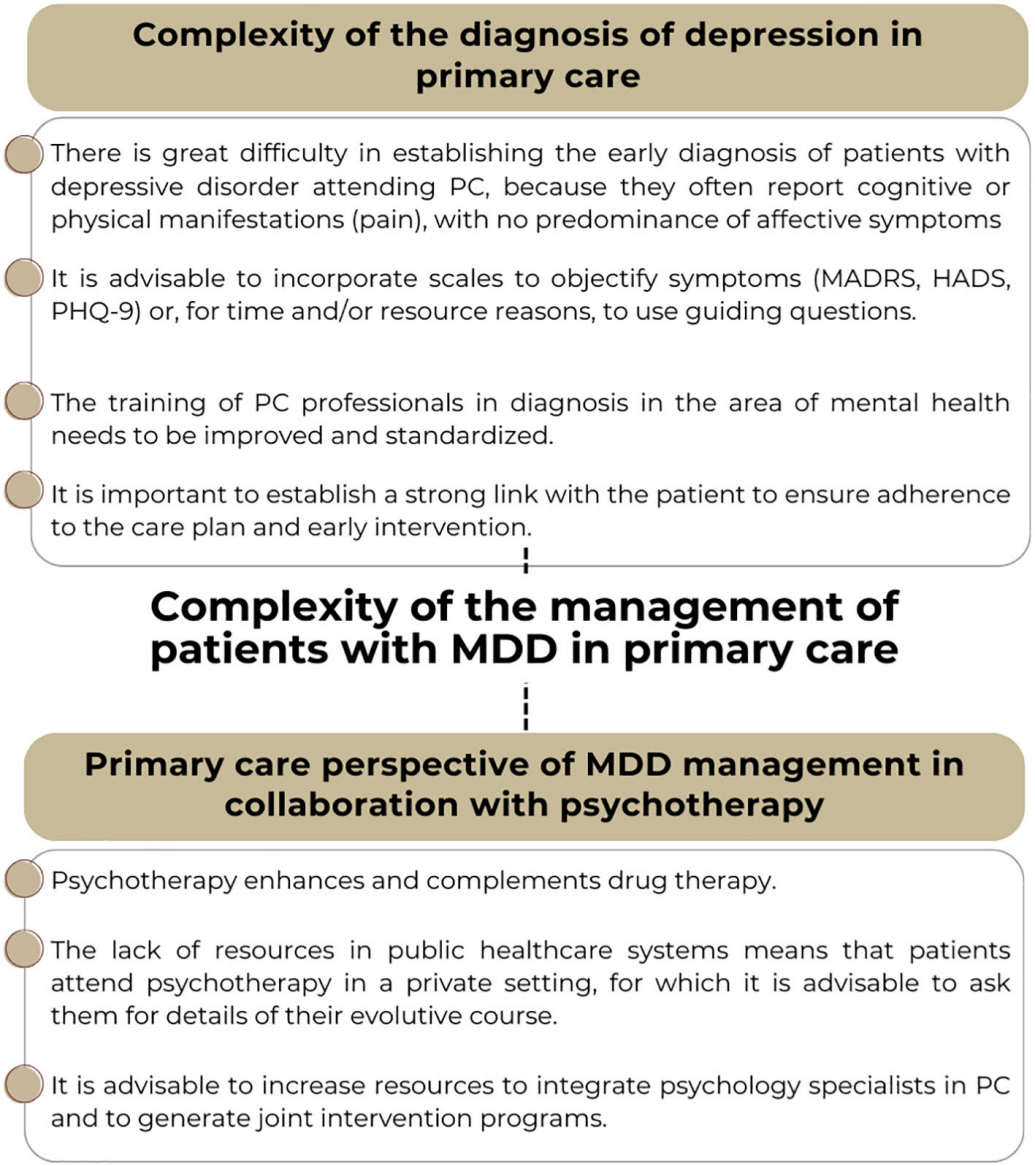
Recommendations and issues to consider regarding the diagnosis of major depressive disorder (MDD) and joint management with a mental health specialist, from the primary care perspective. PC, Primary Care; HADS, Hospital Anxiety and Depression Scale; MADRS, Montgomery-Asberg Depression Rating Scale; PHQ-9, Patient Health Questionnaire-9.

The diagnosis of depression in the PC setting presents significant challenges that require special consideration (**Figure 1**). The difficulty in establishing an early diagnosis is related at least in part to that fact that some patients with depressive disorder attending PC present cognitive or physical manifestations (pain), with no predominance of affective symptoms. This variability in the presentation of symptoms may lead to a delay in diagnosis and the start of the most adequate treatment, as well as to errors in diagnosis (4–6). In this regard, depression remains an underdiagnosed disorder in PC, with only approximately 40% of all patients receiving the necessary treatment (5). The fact that, according to different estimates, depression presents with complaints of psychological distress in 45% of the cases, with complaints of somatic discomfort (fatigue, joint pain or vague pain) in 36%, and organic comorbidities in 19%, means that emotional, behavioral, cognitive and somatic symptoms should be jointly assessed in diagnosing the disorder (7, 8).

To address this complexity, it is crucial to incorporate standardized assessment tools into clinical practice. Thus, there is a need to incorporate scales to objectify symptoms, suggesting the use of guiding questions in cases where, due to time or resource issues, the complete assessment tools cannot be used. Diagnostic scales such as the Hamilton Scale (9), the Montgomery-Asberg Rating Scale (MADRS) (10), the Patient Health Questionnaire (PHQ-9) (11), among others, may provide an objective assessment of the symptoms. However, the reality of the PC setting often makes it difficult to administer patient-reported outcome scales or questionnaires, which, although widely known by healthcare professionals, are not used due to time and resource constraints (12). However, some of the most widely used scales for the diagnosis of MDD require specialized training and are generally more specific for use in mental health settings. In primary care, simpler instruments are needed-tools that do not require specific training and can be self-administered by patients, such as the previously mentioned PHQ-9 and the Hospital Anxiety and Depression Scale (HADS) (13).

In addition to the assessment scales, continuous training of PC professionals in mental health is essential. In this regard, the general perception is that it should be greater in general practitioners in this area. Studies conducted in Spain have shown that 40.6% of the general practitioners considered their training in the management of patients with depression through the use of psychotropic drugs to be insufficient, and the vast majority (92.5%) considered their participation in training activities in this field to be necessary (14). These data are in line with similar analyses conducted at international level, evidencing that 25% of the general practitioners were not comfortable in being able to provide adequate treatment for patients with depression (15). This perception of insufficient training may negatively impact confidence and the management of depression in PC, even more so in a setting where a strong link must be established with the patient to ensure adherence to the care plan and to facilitate early and effective intervention (16). In addition, a substantial number of patients are reluctant to receive treatment for the management of depressive disorder, even after having accepted the diagnosis. Both the causes to which patients attribute the origin of the disorder, and negative beliefs about antidepressants, as well as fear of addiction or adverse effects, can account for such reluctance (17–20).

## Management of MDD patients in PC through treatment with vortioxetine

Various categories of antidepressant drugs have been commonly used in the treatment of MDD, including selective serotonin reuptake inhibitors (SSRIs), serotonin norepinephrine reuptake inhibitors (SNRIs), and tricyclic antidepressants, among others (21). However, approximately half of all patients do not achieve remission of the depressive episode with first-line treatment, so there is considerable debate about its effectiveness (21). Thus, the search for new antidepressants that offer greater effectiveness and tolerability in the management of MDD is essential.

The multimodal antidepressant vortioxetine is a first-line treatment option that directly modulates different serotonin receptors and inhibits the serotonin transporter, affording efficacy in adults comparable to that of most other antidepressants (21–30). Positive results in controlled clinical trials and meta-analyses of these trials have been corroborated in real-life studies (31).

Vortioxetine has a unique multimodal mechanism of action. Its pharmacodynamic profile includes: 1) Inhibits serotonin transporters (SERT), increasing serotonin (5-HT) levels, though with lower transporter occupancy than most SSRIs; and 2) It acts directly on several serotonin (5-HT) receptor subtypes (32). Through its serotonergic actions, vortioxetine indirectly modulates other neurotransmitter systems, including norepinephrine, dopamine, acetylcholine, and histamine, particularly in brain regions relevant to mood and cognition (33). These pharmacodynamic properties are associated with antidepressant efficacy, improvement in cognitive symptoms, and a lower risk of certain side effects such as emotional blunting and sexual dysfunction compared to conventional SSRIs (32).

### Key benefits and practical considerations in PC

#### Dosing flexibility

**Figure 2:**
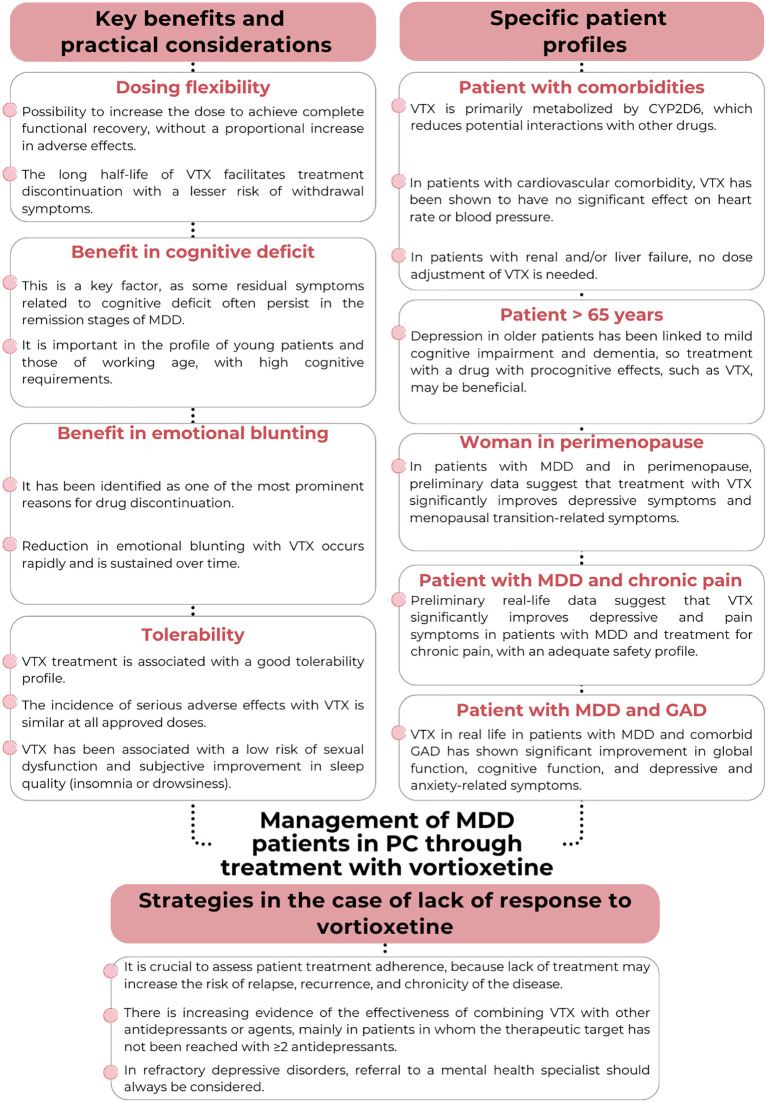
Recommendations and considerations regarding the management of patients with MDD in PC through treatment with vortioxetine. PC, Primary care; GAD, Generalized anxiety disorder; MDD, Major depressive disorder; VTX, Vortioxetine.

Treatment with vortioxetine allows dosing flexibility, since the dose can be increased to achieve complete functional recovery, without a proportional increase in adverse effects (**Figure 2**) (34, 35). Clinical trials and systematic reviews have shown a dose-response relationship with vortioxetine, in contrast to other antidepressants. In this regard, a dose of 20 mg/day is related to greater efficacy than a 10 mg/day dose, without increasing the adverse effects (34, 35). The assessment of tolerability at 15 days after treatment initiation and of response at 2–4 weeks is advised, with dose adjustments if needed (22). The half-life of vortioxetine facilitates treatment discontinuation with a lesser risk of withdrawal symptoms (6, 22, 32). Discontinuation symptoms (DS) with vortioxetine are rare, occurring in only 3% of patients who stopped treatment (36). DS is more likely when vortioxetine is stopped accidentally and without medical advice. The risk of DS is not related to age, sex, discontinuation method, dose, comorbidities, polytherapy, or treatment duration (36). This adaptation of treatment is a key point in the management of MDD with antidepressants, particularly from the perspective of PC, because it allows for avoiding frequent changes in drug. A high frequency of changes in medication may cause uncertainty and anxiety in patients, decrease confidence in the treatment and lead to lower long-term adherence, as well as create treatment resistance and modify the course of depression (20, 37, 38).

Recent analyses also suggest that higher doses of vortioxetine are associated with a more pronounced reduction in depressive symptoms, a faster onset of therapeutic effects, greater improvement in anxiety symptoms, increased effectiveness in patients with a history of trauma, and more substantial alleviation of specific symptoms such as anhedonia and anergy (39–43).

#### Benefit in the treatment of cognitive deficits

Vortioxetine has shown benefits in the treatment of cognitive deficits, which tend to be common in patients with depression, both in controlled clinical trials and in real-life studies (**Figure 2**) (31, 44–46). This is a relevant factor in the functional recovery of the patient, since some symptoms related to cognitive deficit (mainly attention, memory and processing speed) are residual symptoms that often persist in the remission phases of MDD (47). Specifically, this is an important aspect in the profile of young patients and those of working age, with high cognitive requirements.

Improvement of cognitive function is also a particularly relevant issue in the elderly population, with cognitive changes associated with age and/or with Alzheimer’s disease, where treatment with vortioxetine may have important therapeutic implications, given the need for effective therapies that address both the depressive symptoms and cognitive impairment (48, 49). In addition, different studies have addressed the treatment and improvement of cognitive function in specific patient groups, such as patients with MDD after stroke or with Parkinson’s disease (50, 51).

#### Benefit in terms of emotional blunting as an adverse effect of treatment with antidepressants

Emotional blunting is a common adverse effect reported by patients with MDD who are receiving antidepressant treatment, particularly SSRIs and SNRIs (52). This phenomenon is characterized by a reduced ability to experience emotions, both positive and negative; feelings of disengagement; and a decreased ability to respond emotionally. It is estimated that 40-60% of all patients treated with SSRIs or SNRIs experience some degree of emotional blunting (53). It has been identified as one of the most prominent reasons for drug discontinuation, and in response, clinicians often consider reducing the dose of antidepressant treatment or switching to another treatment in an attempt to control this effect (**Figure 2**) (53).

Based on real-life results, treatment with vortioxetine 5–20 mg effectively improves the emotional blunting associated with antidepressant treatment in patients with MDD and an inadequate response to SSRIs/SNRIs (54, 55). Such recovery is accompanied by improvement in the core of symptoms associated with depression, sleep duration and quality, motivation, and cognitive and global functioning of the patient (54, 55). Furthermore, the reduction in the severity of emotional blunting after vortioxetine initiation occurs rapidly and is sustained over time (54).

#### Tolerability and perception of treatment safety

Tolerability is one of the key aspects to ensure adherence to antidepressant treatment in MDD (20). Treatment with vortioxetine has a good tolerability profile, as clinical trials and real-life studies have shown that the incidence of adverse effects such as weight gain, insomnia and sexual dysfunction, and the cardiovascular safety profile, are comparable to placebo or other antidepressants (**Figure 2**) (24, 30, 31, 35). Broad clinical profile or vortioxetine and high level of safety are supported by evidence from randomized trials, post-marketing and case studies, including populations with epilepsy, elderly patients, people with dementia, and those with somatic comorbidities (39, 56–58). In fact, real-life data estimate a high acceptability and tolerability of treatment, with low discontinuation ratios, according to the conclusions of a recently published meta-analysis (31).

As discussed above, treatment with vortioxetine allows for the possibility of increasing the dose without a proportional increase in adverse effects (34, 35). A systematic review of clinical trials and real-life studies focused on vortioxetine indicated that the incidence of serious adverse effects was 2.9% for the 5–10 mg dose and 2.2% for the 15–20 mg dose (33). This is an important advantage, because the most commonly used second-generation antidepressants (citalopram, escitalopram, fluoxetine, paroxetine, sertraline, venlafaxine and mirtazapine) have an optimum balance of efficacy, tolerability and acceptability only in the lowest ranges of their doses approved in the Summary of Product Characteristics (59). Thus, it is estimated that the discontinuation rate of these drugs due to adverse effects increases sharply as the dosage is increased (59).

As an exception, it should be noted that treatment with vortioxetine 5–15 mg has been associated with a low risk of treatment-related sexual dysfunction, while the 20 mg dose was associated with a slight increase of this risk (22, 32).

Antidepressants have been reported to exert different effects on sleep architecture; in this regard, some treatments (e.g., SSRIs) can eventually lead to sleep disturbances, which may exacerbate the depressive symptoms (60, 61). This may help to create a vicious cycle, as it may lead to an increase in the dose of the antidepressant or the addition of drugs to improve sleep quality (62). In contrast, data on improvement of subjective sleep quality during treatment with vortioxetine, which does not usually cause insomnia or drowsiness, are found in the literature (63, 64).

### Strategies in the case of lack of response to vortioxetine monotherapy

The response and remission rates in patients with MDD treated with vortioxetine in real life have been estimated to be 66.4% (95%CI = 51.2-81.5%) and 58% (95%CI = 48.9-67.1%), respectively. It is very important to assess patient adherence to therapy, because poor adherence has an impact on the recovery process and may increase the risk of relapse (**Figure 2**) (37, 38). In the event of lack of response to antidepressant treatment, the current guidelines on the management of MDD in PC recommend a number of strategies, including: i) increasing or optimizing the dose of the drug being used; ii) switching to another antidepressant; iii) combining antidepressants with different profiles; and iv) enhancing the antidepressant with other pharmacological agents (6). In addition, in depressive disorders refractory to treatment, referral to a mental health specialist should be considered (6).

There is growing evidence of the effectiveness of combining vortioxetine with other antidepressants or agents, mainly in patients in whom the therapeutic target has not been reached with ≥ 2 antidepressants. In general, the combination of vortioxetine and bupropion (a potent inhibitor of cytrochrome P4502D6) may be a useful and well-tolerated option, though it is underused in the PC setting. The association of vortioxetine with a slow metabolizer such as bupropion requires dose adjustment of both drugs, due to the potential incidence of certain side effects that do not appear when either drug is used alone (65, 66).

Mirtazapine is presented as a more attractive option for combined antidepressant therapy, being easy to use in the primary care setting (67). Its pharmacodynamic profile effectively complements most antidepressants, offering additional advantages (6). However, it is important to consider that mirtazapine involves an increased risk of weight gain and metabolic alterations (68). Therefore, careful and continuous assessment of its long-term tolerability is recommended in each patient in order to control potential cardiovascular risk factors.

Similarly, the addition of trazodone to vortioxetine therapy may be considered. Recent studies have demonstrated the strong clinical effectiveness of trazodone (69), and, when used at appropriate doses, this combination does not present a significant risk of serotonin syndrome. As regards vortioxetine augmentation therapy with other agents such as lithium salts or antipsychotics (practices more common in psychiatry than in PC), such treatments have been shown to be effective in two ways: on one hand, they improve resistant depressions, and on the other hand, they offer the advantage of including vortioxetine versus other antidepressants for the treatment of MDD with other comorbidities (66, 67).

### Specific patient profiles

#### Patients with concomitant disorders or polymedicated individuals

Antidepressant therapies, particularly SSRIs and SNRIs, interact with other drugs, mainly through cytochrome P450 (CYP450) inhibition (70). This mechanism may lead to increased or decreased levels of other drugs in patients with concomitant diseases requiring drug treatment, causing toxicity. The above is particularly important in patients with MDD and cardiovascular diseases, renal and/or liver failure, diabetes or chronic pain (70–73). In these cases, vortioxetine would be recommended as the first option, since it offers advantages thanks to its pharmacological profile (6). Vortioxetine is metabolized in the body more simply than other antidepressants, mainly through CYP2D6, without affecting (inhibiting or inducing) the different cytochrome P450 isoenzymes involved, thus reducing potential interactions with other drugs (**Figure 2**) (32, 74). Clinical pharmacokinetic studies have shown that co-administering vortioxetine with strong CYP2D6 inhibitors such as bupropion, fluoxetine, or paroxetine leads to approximately a two-fold increase in vortioxetine exposure (AUC and Cmax) (75). Therefore, it is recommended to reduce the vortioxetine dose by half when used alongside strong CYP2D6 inhibitors to minimize the risk of concentration-dependent side effects. Specifically, in patients with cardiovascular comorbidity, vortioxetine has been shown to have no significant effect on heart rate or blood pressure (74). In patients with renal and/or liver failure, no dose adjustment of vortioxetine is required, unlike with other antidepressants (6, 76–78).

#### Patients over 65 years of age

As previously commented, vortioxetine shows significant improvement of outcomes in real-life and clinical trials in relation to the cognitive symptoms associated with MDD, which may be relevant in the elderly population. Depression in older patients has been linked to mild cognitive impairment and dementia, so treatment with a drug with procognitive effects may be beneficial (**Figure 2**) (79–81). Preliminary and specific studies on this patient profile demonstrated clinically significant improvement in the depressive symptoms, cognitive performance, functional recovery and quality of life of patients with MDD and cognitive impairment, including early-stage Alzheimer’s disease (49, 82).

#### Females of childbearing potential and in the perimenopausal phase

There are limited human data on the use of vortioxetine during pregnancy, but animal studies have shown reproductive toxicity. For this reason, the recommendation is to administer this medication during pregnancy only if the benefits outweigh the potential risks to the fetus (22). With regard to lactation, data from studies in animals show vortioxetine to be excreted in milk, and the same is expected to occur in humans. The Relative Infant Dose (RID) index for vortioxetine is low (less than 2%) so the risk of exposure of the child to significant doses of the drug received in milk is small (83). However, until more data are available, vortioxetine should be used with careful infant monitoring during breastfeeding. Menopause is characterized by a complex interaction of biological, psychological and social factors. The hormonal changes in menopause, particularly estrogen reduction, affect mood and mental health, and depression is an important concern due to its high prevalence in these patients (84, 85). In the case of patients with MDD and in transition to menopause, preliminary data have indicated that vortioxetine treatment not only significantly improves depression-related symptoms and indicators (with response and remission rates of 75% and 70.8%, respectively), but also improves menopause-related symptoms (**Figure 2**) (86).

#### Patients with chronic pain

Chronic pain is a state of stress in which depression becomes one of the most common psychiatric complications in the affected individual (87). Depression in patients with chronic pain is associated with a reduction in pain threshold, an increase in pain perception, and a poorer response to analgesics (87). In addition, the presence of pain is related to more severe depressive symptoms in patients, including sleep disturbances, increased anxiety, and cognitive deficit (88). Preliminary data obtained in routine clinical practice in patients treated for chronic pain and diagnosed with MDD who had not previously responded to other antidepressants show that vortioxetine significantly improves depressive and pain symptoms, with an adequate safety profile (**Figure 2**) (89). Recent studies have also demonstrated that vortioxetine can effectively treat neuropathic pain and has shown efficacy in the management of patients with burning mouth syndrome (90, 91).

#### Patients with MDD and generalized anxiety disorder

Comorbidity of MDD and generalized anxiety disorder (GAD) is common, and there is evidence that patients with depressive disorders are 11.7 times more at risk of developing GAD than patients without depressive symptoms (92–94). Furthermore, the comorbid presence of GAD increases the risk of relapse of the depressive disorder (**Figure 2**) (95). The multimodal mechanism of action of vortioxetine, at the level of the serotonergic pathways, could make vortioxetine a tool for the treatment of patients with MDD and generalized anxiety disorder (96, 97). In fact, the use of vortioxetine in real life in a subgroup of patients with MDD and comorbid GAD has shown significant improvement in global function, cognitive function, and depressive and anxiety-related symptoms (98). In addition, good tolerability is observed at the standard doses (5–20 mg) (98).

### Primary care perspective of MDD management with vortioxetine in combination with non-pharmacological therapy

Psychotherapy enhances and complements drug therapy from the mildest stages of MDD (**Figure 1**) (6). However, the options for patient referral from PC to the psychotherapy services vary among the different regions (Autonomous Communities) of the country, and in general, in the context of the public healthcare system, there are no facilities for making such referrals. In the context of public health, few resources are available in terms of professionals specializing in psychology in PC, and their activity is limited (99). In fact, in Spain the rate is 5.71 clinical psychologists/100,000 inhabitants, with waiting times for the first visit of approximately 200 days (100). Demand from PC therefore would be aimed at securing greater coordination with the specialized services, since failure to do so may lead to a deficit in the detection of mental disorders, and particularly of MDD (101). Several proposals for the integration into PC teams of specialists in psychology have been developed in this regard - the results of which suggest that treatment is up to three times more effective than if patient management is only carried out by the general practitioner (102, 103). The main consequence of these integration programs is an increase in the percentage of patients who recover (102, 103).

## Future research and clinical practice

Recent evidence highlights the efficacy and tolerability of vortioxetine in MDD and its potential benefits in cognitive symptoms. However, important gaps persist that require further research and focused enhancements.

First, most available data derive from randomized clinical trials conducted under conditions which may not fully represent real-world patient populations. Future studies should prioritize real-world research to better understand effectiveness and safety of vortioxetine across diverse clinical settings (31).

There is also a need to clarify the management of adverse events in special populations, such as those with complex psychiatric or neurological comorbidities (104). Finally, mechanistic studies exploring the drug’s multimodal effects could inform personalized treatment approaches and the development of combination therapies.

Addressing these gaps will help optimize the clinical use of vortioxetine and expand its therapeutic potential.

## Conclusions

In conclusion, vortioxetine is a very valuable treatment option in the management of MDD in PC. Its multimodal mechanism of action offers significant advantages, including dosing flexibility, benefits in the treatment of cognitive deficits, and an improvement of the emotional blunting normally associated with other antidepressants. The good tolerability of vortioxetine, with a low incidence of adverse effects, favors treatment adherence. In addition, its effectiveness in the management of cognitive symptoms positions it as a particularly relevant option for patients of working age and older adults. Vortioxetine represents an important tool in the therapeutic armamentarium of the general practitioner, offering an alternative that can significantly improve the quality of life of patients with depression.

## Data Availability

The original contributions presented in the study are included in the article/supplementary material. Further inquiries can be directed to the corresponding author.
